# Isolation and characterization of cellulose-mineralizing haloalkaliphilic bacteria from Siberian soda lakes

**DOI:** 10.3389/fmicb.2024.1523074

**Published:** 2024-12-23

**Authors:** Dimitry Y. Sorokin, Alexander Y. Merkel, Tatjana V. Khizhniak

**Affiliations:** ^1^Winogradsky Institute of Microbiology, Federal Research Centre of Biotechnology, Russian Academy of Sciences, Moscow, Russia; ^2^Department of Biotechnology, Delft University of Technology, Delft, Netherlands

**Keywords:** cellulose, cellulotrophic bacteria, haloalkaliphilic, soda lakes, cellulase

## Abstract

Soda lakes are unique double-extreme habitats characterized by high salinity and soluble carbonate alkalinity, yet harboring rich prokaryotic life. Despite intensive microbiology studies, little is known about the identity of the soda lake hydrolytic bacteria responsible for the primary degradation of the biomass organic matter, in particular cellulose. In this study, aerobic and anaerobic enrichment cultures with three forms of native insoluble cellulose inoculated with sediments from five soda lakes in south-western Siberia resulted in the isolation of four cellulotrophic haloalkaliphilic bacteria and their four saccharolytic satellites. The final aerobic enrichment included a cellulotrophic bacteroidetes (strain ABcell3) related to *Sporocytophaga* accompanied by a hemicellulolytic *Marinimicrobium* strain ABcell2. The anaerobic enrichments resolved in three primary cellulotrophic bacteria and their three saccharolytic bacteroidetes satellites. The culture selected on amorphous cellulose (ac) included a new cellulotrophic member of the *Chitinispirillaceae* (*Fibrobacterota*)—strain ANBcel5, and two different saccharolytic satellites from the *Marinilabiliales* and *Balneolales* orders. The final enrichment selected on Sigma 101 cellulose consisted of an endospore-forming cellulotrophic strain ANBcel31 belonging to the genus *Herbivorax* (*Acetivibrionales*) and its saccharolytic satellite from the *Balneolales* order. The anaerobic enrichment on a filter paper yielded a binary consortium with the cellulotrophic endospore-forming *Halanaerobiales* strain ANBcel28 in obligate syntrophy with a cellobiose-utilizing *Natronincola*. A functional genome analysis of the cellulotrophic isolates confirmed the presence of a large repertoire of genes encoding excreted cellulases, mostly from the GH9 and GH5 families, and indicated that in the endospore-forming anaerobic strains, ANBcel28 and ANBcel31 most of their endo-glucanases are assembled in cellulosomes. Overall, this study showed that cellulose can be mineralized in soda lakes at moderately saline and highly alkaline conditions either by aerobic or fermentative haloalkaliphilic bacteria.

## Introduction

Soda lakes are unique inland saline habitats, characterized by brines with extremely high and stable pH values (ranging from 9 to 11) due to the presence of sodium carbonates at molar concentrations. This provides a strong alkaline buffering capacity of up to several mols l^−1^, which is mostly suitable only for a specialized group of prokaryotic extremophiles adapted both to high salt and high alkalinity called haloalkaliphiles or natronophiles (Grant and Jones, 2016). In the past 30 years, the soda lake prokaryotic communities attracted much interest from general microbiology and biotechnology prospects and have been intensively studied both by cultivation and culture-independent molecular approaches, making them one of the best microbiologically characterized saline systems (Fujinami and Fujisawa, 2010; Sarethy et al., 2011; Sorokin et al., 2014, 2015a,b; Sorokin, 2017; Uma et al., 2020; Vavourakis et al., 2018; Zorz et al., 2019).

Despite being double-extreme habitats, many soda lakes, even hypersaline, have high primary production of organic matter (Haines et al., 2023; Oduor and Schagerl, 2007; Samylina et al., 2014) fueling active microbial carbon cycling, including its mineralization. The key limited stage of the latter process consists of enzymatic hydrolysis of biopolymers, a large part of which includes cellulose and hemicelluloses. Furthermore, wind-carried plant detritus can enter such lakes from the surrounding lands.

So far, little is known about the identity of prokaryotes involved in cellulose mineralization in soda lakes. However, recent progress has been made, particularly with the focus on extremely halophilic natronoarchaea that are abundant in hypersaline soda lakes. This resulted in the discovery of the first natronoarchaeal genus *Natronobiforma* with a prominent potential for the utilization of native celluloses as a growth substrate and to prove a similar, albeit less active, capacity in the known natronarchaeal species *Natronolimnobius baerhuensis* (Sorokin et al., 2015a,b, 2018). Genome analysis of these natronoarchaea confirmed the presence of multiple copies of marker genes encoding endo-*β*-1,4-glucanases from the GH5 and GH9 families implicated in primary cellulose depolymerization (Elcheninov et al., 2023). However, hypersaline soda lakes with the domination of natronoarchaea are very rare, while most others belong to the low to moderately saline category dominated by bacteria. There is a definite gap of knowledge regarding the identity of soda lake haloalkaliphilic bacteria with the potential to mineralize native celluloses. The only such verified example known so far is a low-salt tolerant anaerobic cellulosome-forming *Clostridium alkalicellulosii* (currently reclassified as *Herbivorax alkalicellulosii*) isolated from a soda lake Hadyn in Tuva Republic (Zhilina et al., 2005; Zvereva et al., 2006).

The current study was aimed to fill the abovementioned knowledge gap resulting in the isolation and characterization of four cellulose-utilizing haloalkaliphilic bacteria from Siberian soda lakes representing new taxa in the phyla *Bacteroidota* (order *Cytophagales*), *Fibrobacterota* (order *Chitinispirillales*), and *Bacillota* (orders *Acetivibrionales* and *Halanarobiales*).

## Materials and methods

### Inoculum

Sediment samples with near-bottom brines (1:1 v/v) were collected from five soda lakes in the southern part of Kulunda Steppe (Altai region, Russia) between July 2020 and 2021 (N52^o^06’/ E79^o^09’; N51^o^37’/ E79^o^50’; N51^o^39’/ E79^o^48’; N51^o^40’/ E79^o^54’- E79^o^54’). The total salt concentration of the brines ranged from 40 to 200 g l^−1^, the pH from 9.7 to 10.5, and the carbonate alkalinity from 0.4 to 2.0 M. The brines’ temperature during sampling was 25–28°C. For aerobic enrichment, the upper 1 cm of oxic sediments was sampled, and for anaerobic cultivation, anoxic sulfidic sediment from a 5 to 15 cm layer was taken by a corer. The two types of samples from individual lakes were combined in equal proportions to form two “master mixes” used as inocula.

### Enrichments and isolation of cellulotrophic bacteria

The enrichment medium, based on sodium carbonate/bicarbonate buffer, contained either 1 M total Na^+^, with 0.1 M Na^+^ from NaСl and 0.9 M Na^+^ from carbonate–bicarbonate, and had a final pH of 9.5. The base medium was sterilized at 120°C for 20 min and further supplemented with 1 mL each of trace metal and vitamin mix solution (Pfennig and Lippert, 1966), 1 mM MgCl_2,_ and 20 mg L^−1^ of yeast extract. Aerobic enrichments were incubated in 100 mL screw-cap bottles with rubber septa and 20 mL volumes on a rotary shaker at 150 rpm and 37°C. Anaerobic cultivation was performed in 115 mL serum bottles with black rubber stoppers and 80 mL of medium. Anaerobiosis was achieved using several cycles of sterile argon gas flushing-evacuation and final reduction of the medium—by adding 0.2 mM Na_2_S solution. The bottles were incubated at 37°C statically with shaking twice a day. Three types of insoluble cellulose [sterilized as a 5% (w/v) suspension] were used as substrates at a final concentration of 1 g L^−1^: amorphous cellulose (ac) prepared from Avicel, as described previously (Sorokin et al., 2015a,b); filter paper (fp) mesh from shredded Whatman filters; and Sigma celluloses 101 (Sigma-Aldrich). The incubations were monitored for visible signs of cellulose degradation and bacterial growth by phase contrast microscopy (Zeiss Axioplan Imaging 2 microscope, Göttingen, Germany). In addition, H_2_ and methane were monitored in the gas phase of anaerobic incubations using gas chromatography. Positive primary enrichments were then serially diluted up to 10^10^ in the same medium containing the original cellulose forms in 23 mL serum bottles with 5 and 10 mL medium for aerobic and anaerobic cultivation, respectively, and maximum positive dilutions (10^8^–10^9^) were used either for further purification in liquid dilution series or by soft agar-shake plating (using washed Daishin agar, final concentration 1% w/v) with ac as substrate, enabling easy detection of cellulolytic colonies (Sorokin et al., 2015a,b). Aerobic plates were incubated in plastic bags to prevent drying, while for anaerobic cultures the plates were prepared from anaerobic medium and incubated in gas jars filled with argon and supplied with the O_2_-scavenging catalyzer (both from Oxoid). In case of positive colony formation with clearance of ac, those were placed into liquid medium with ac, and the cycle from liquid medium to plating was repeated until the formation of uniformed colony morphotype was achieved. Furthermore, when a stable association of primary cellulotrophs with a non-cellulolytic satellite(s) (not forming ac-clearance zones and not growing with ac in liquid culture) was achieved, the latter were also isolated in pure culture by using soft agar-shake plating with cellobiose.

### Genome sequencing

Genomic DNA was obtained from cellulose-free cells grown in liquid culture using the FastDNA™ SPIN Kit for Soil (MP Biomedicals, United States). Shotgun WGS libraries were prepared using the KAPA HyperPlus Library Preparation Kit (KAPA Biosystems, UK). The genome sequencing was performed using the Illumina NextSeq 550 or NovaSeq 6,000 system (Illumina, San Diego, CA, United States). The genome assemblage was performed using Unicycler v.0.5.0 (Wick et al., 2017) and automatically annotated using the PGAP (Tatusova et al., 2016) in GenBank.

### Phylogenomic analysis

Phylogenomic reconstructions were based on the alignment of 120 single-copy conserved bacterial marker proteins according to the Genome Taxonomy Database (release GTDB-Tk v2.4.0) (Chaumeil et al., 2022; Rinke et al., 2021). Apart from the cultured bacteria, MAGs closely related to our isolates were also included in the analysis, with more than 90% completeness and less than 5% contamination, determined using CheckM (Parks et al., 2015) and preferably originated from haloalkaline habitats. The aligned sequences were trimmed using trimAl 2.rev0 build 2019-08-05 with “-automated1” (optimized for Maximum Likelihood phylogenetic tree reconstruction) and “-gt 0.96” modes (Capella-Gutiérrez et al., 2009), resulting in 22,097 aa length alignment. The trees were built using the IQ-TREE2 program v2.2.0.3 (Minh et al., 2020) with fast model selection via ModelFinder (Kalyaanamoorthy et al., 2017) and ultrafast bootstrap approximation (Minh et al., 2013) as well as approximate likelihood-ratio test for branches (Anisimova and Gascuel, 2006). Relative evolutionary divergence (RED) was calculated according to Parks et al. (2018) and the bac120_r220 tree from the GTDB repository.^1^

For whole-genome comparison, average amino acid identity (AAI) using the EzAAI v1.1 (Kim et al., 2021) was used. The genome assemblies of four cellulotrophic isolates and their four saccharolytic satellites are deposited in the GenBank under BioProject PRJNA949680. Their accession numbers and genome statistics are shown in Supplementary Table S1.

### Functional genome analysis

CAZymes genes (glycoside hydrolases and polysaccharide lyases of the GH and PL families, respectively) were first screened in the genomes using dbCAN3 (Zheng et al., 2023) and then verified by using UniProt Blast and HHMER_pfam (for domain architecture). Enzyme localization was predicted using SignalP v.6.0 (Teufel et al., 2022).

### Analyses

H_2_ and CH_4_ formation in the gas phase of anaerobic enrichments were measured on the Chromatec Crystal 5,000 gas chromatograph (Russia), equipped with a HayeSep 80–100 mesh column (2 m x 3 mm) at 40°C, thermal conductivity and flame ionization detectors set at 200°C; an argon gas carrier; and a flow rate of 25 mL min^−1^. Fermentation products in the cell/cellulose-free supernatant of anaerobic cultures were assayed using HPLC with a Bio-Rad HPX-87H column at 60°C. The mobile phase consisted of 1.5 mM H_3_PO_4_, with a flow rate of 0.6 mL min^−1^, and detection was carried out using a UV/Refraction Index Detector (Waters 2,489). For electron microscopy, residual cellulose was allowed to settle, and mostly free bacterial cells were centrifuged, resuspended in 0.5 M NaCl, fixed with paraformaldehyde (final concentration 3%, v/v) for 4 h at 4°C, centrifuged again, resuspended in 0.5 M NaCl, positively contrasted with 1% (w/v) uranyl acetate, and observed under a JEOL 100 electron microscope (Japan).

## Results and discussion

### Enrichment and purification of cellulose-utilizing bacterial consortia from soda lakes

**Figure 1:**
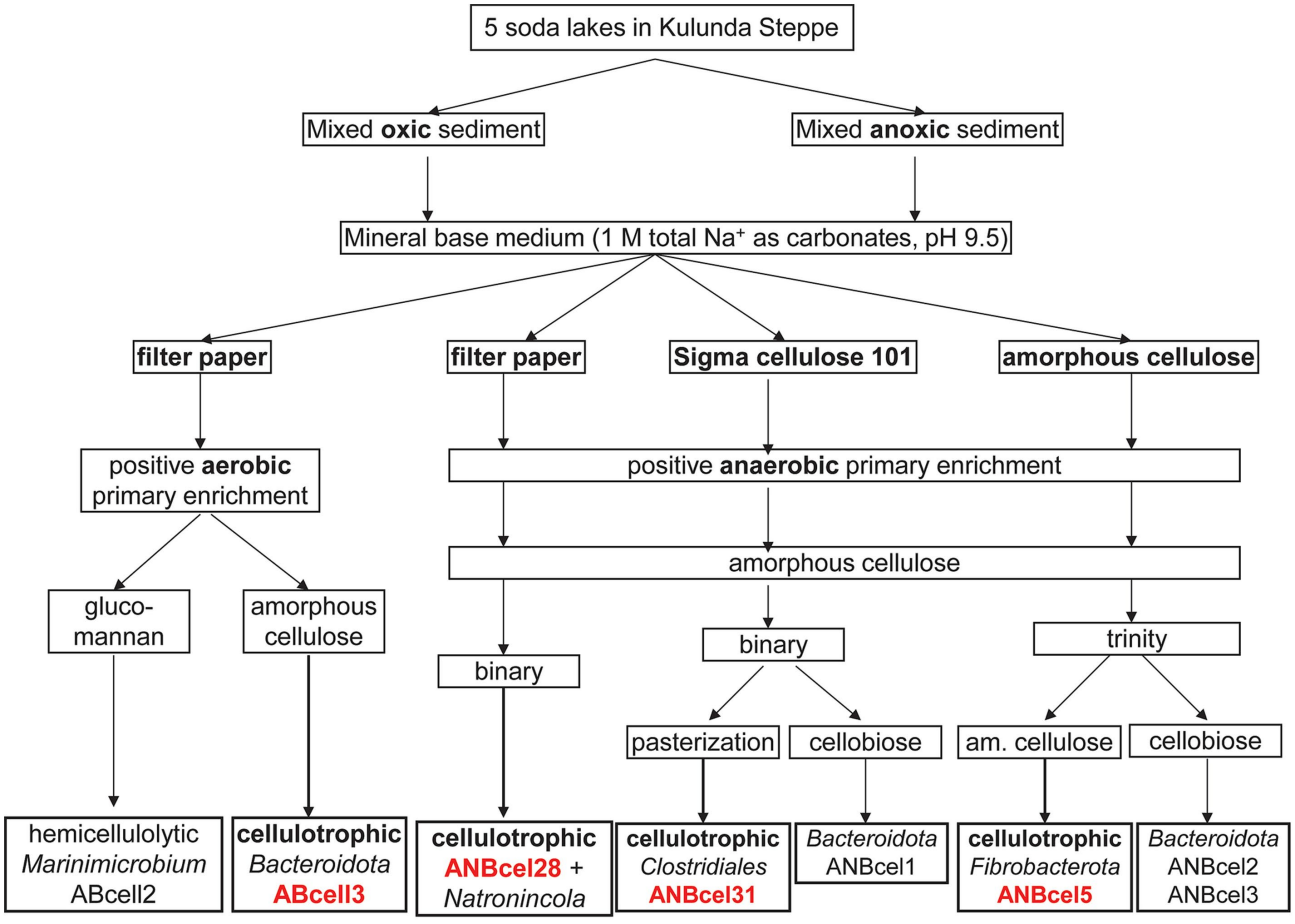
Enrichment and isolation scheme of soda lake bacteria involved in cellulose mineralization.

The general scheme of enrichments and further purification steps of the cellulotrophic bacterial consortia from soda lakes is shown in Figure 1.

**Figure 2:**
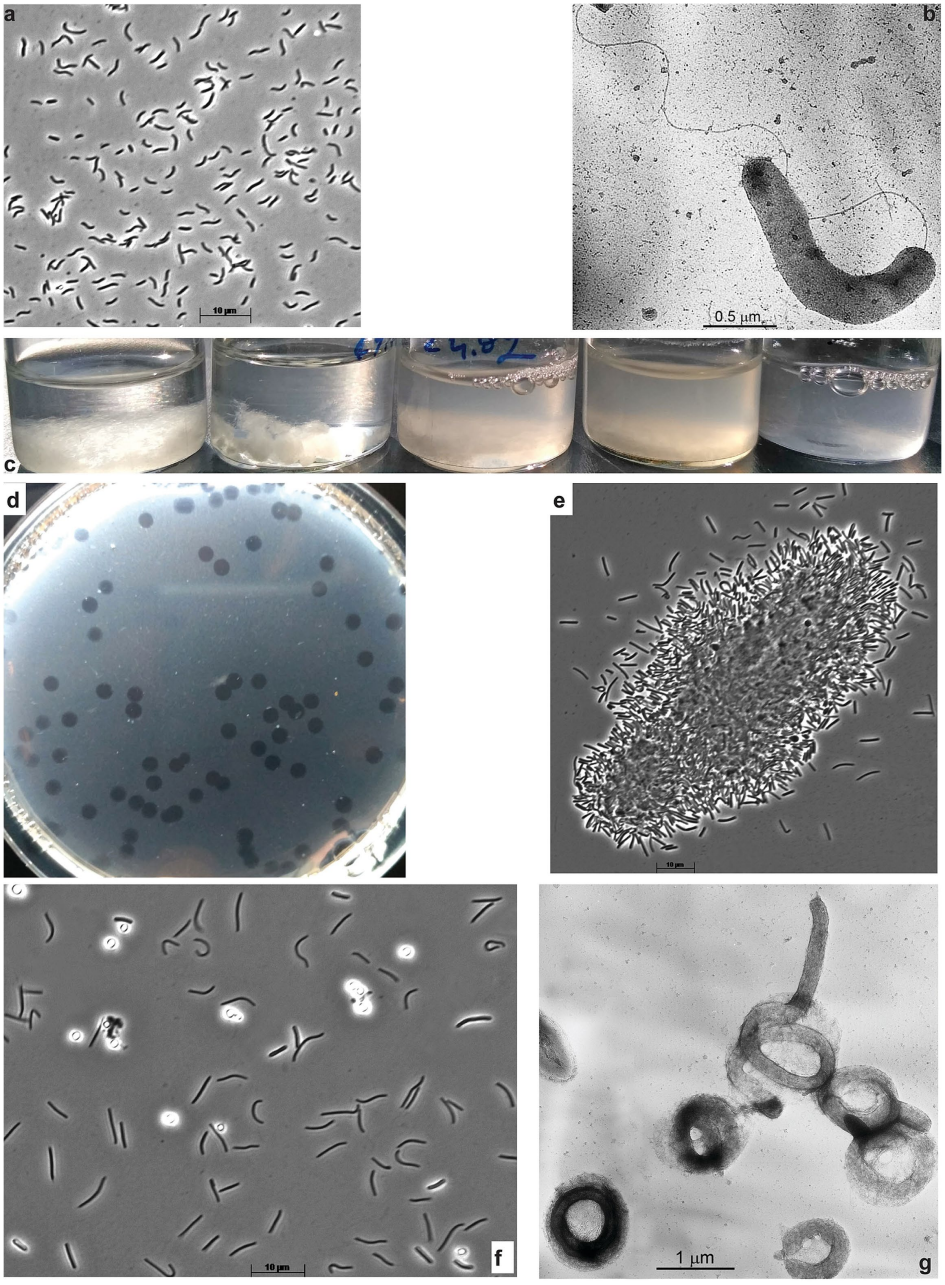
Cell morphology of aerobic hemicellulolytic strain ABcell2 **(A,B)** grown on glucomannan and macro-**(C,D)** and micro **(E–G)** morphology of aerobic cellulolytic strain ABcell3 grown on cellulose. **A,B**,**E,F** – phase contrast and **B,G** – electron microscopy microphotographs. **(C)** Dynamic of filter paper degradation in the culture of strain ABcell3; **(D)** Spot-colonies of strain ABcell3 forming clearance zones on soft-agar-shake plates with amorphous cellulose. **(E)** Colonization of amorphous cellulose particle by cells of strain ABcell3; **(F,G)** Cyst-like circular cells formed by strain ABcell3 during growth on amorphous cellulose.

Aerobic enrichments with ac, fp, and Sigma, 101 types of cellulose all showed similar patterns of progressive colonization of cellulose fragments with various bacterial morphotypes dominated by cyst-like refractive circles within 3 weeks of incubation (Supplementary Figure S1A). Because of the obvious similarity, the cultures obtained with all three forms of cellulose were mixed, and the resulting mixed culture was then subjected to serial dilutions with ac, which produced a positive culture up to (10^8^). At this stage, the culture consisted of mostly two morphotypes: circular cells mentioned above and motile loose vibrio. Further purification attempts on a liquid medium with ac did not result in pure culture isolation (according to the 16S rRNA gene sequencing). However, the vibrio member can be readily separated and purified using glucomannan (Megazyme) as substrate: first in the offshoot into liquid culture followed by surface plating and isolation of pure colonies. Those showed fast growth on glucomannan and cellobiose but were unable to grow on ac, thus representing a satellite of a primary cellulotroph, obviously represented by circular phenotype in the original enrichment on celluloses. The glucomannan-utilizing bacterium was designated strain ABcell2 and was identified by 16S rRNA gene sequencing as a member of the order *Cellvibrionales* in the class *Gammaproteobacteria* (Figures 2A,B). The final selection of a primary aerobic cellulotroph was achieved using the ac-containing soft agar-shake approach, as surface spreading (most often applied for aerobic microbes) yielded no colonies. This resulted in a peculiar phenomenon: the spot-clearance of ac was forming without visible colonial cell growth (Figure 2D) which, most probably, was a result of the cell gliding-motility behavior. When the material from those spots was transferred into the liquid medium with various forms of cellulose, it resulted in bacterial growth and cellulose colonization and degradation (Figures 2C,E). The 16S rRNA gene sequencing from several such cultures confirmed the purity of the isolated cellulotroph designated as strain ABcell3 and identified it as a member of the order *Cytophagales* (*Bacteroidota*). The bacterium has a complex cell morphology, ranging from flexible rods with gliding motility in young cultures to massively produced cyst-like circles in the later stage (Figure 2F), superficially resembling such structures in *Sporocytophaga* (Holt and Leadbetter, 1967). However, the refractive cocci formed by ABcell3 upon closer examination using electron microscopy, apparently have a different nature (see Figure 2G).

The primary anaerobic enrichments on three different forms of cellulose showed intensive colonization of cellulose fragments with domination of refractive coccoids (Supplementary Figures S1B–D), except that the color of ac enrichments remained whitish, while the fp and Sigma 101 cellulose cultures became intensively yellow. Gas phase analysis showed the formation of H_2_ (1–3%) and methane (up to 10%). However, methane ceased to form in further purifying serial dilutions. Further purification demonstrated that the different forms of cellulose selected different primary anaerobic cellulotrophs.

**Figure 3:**
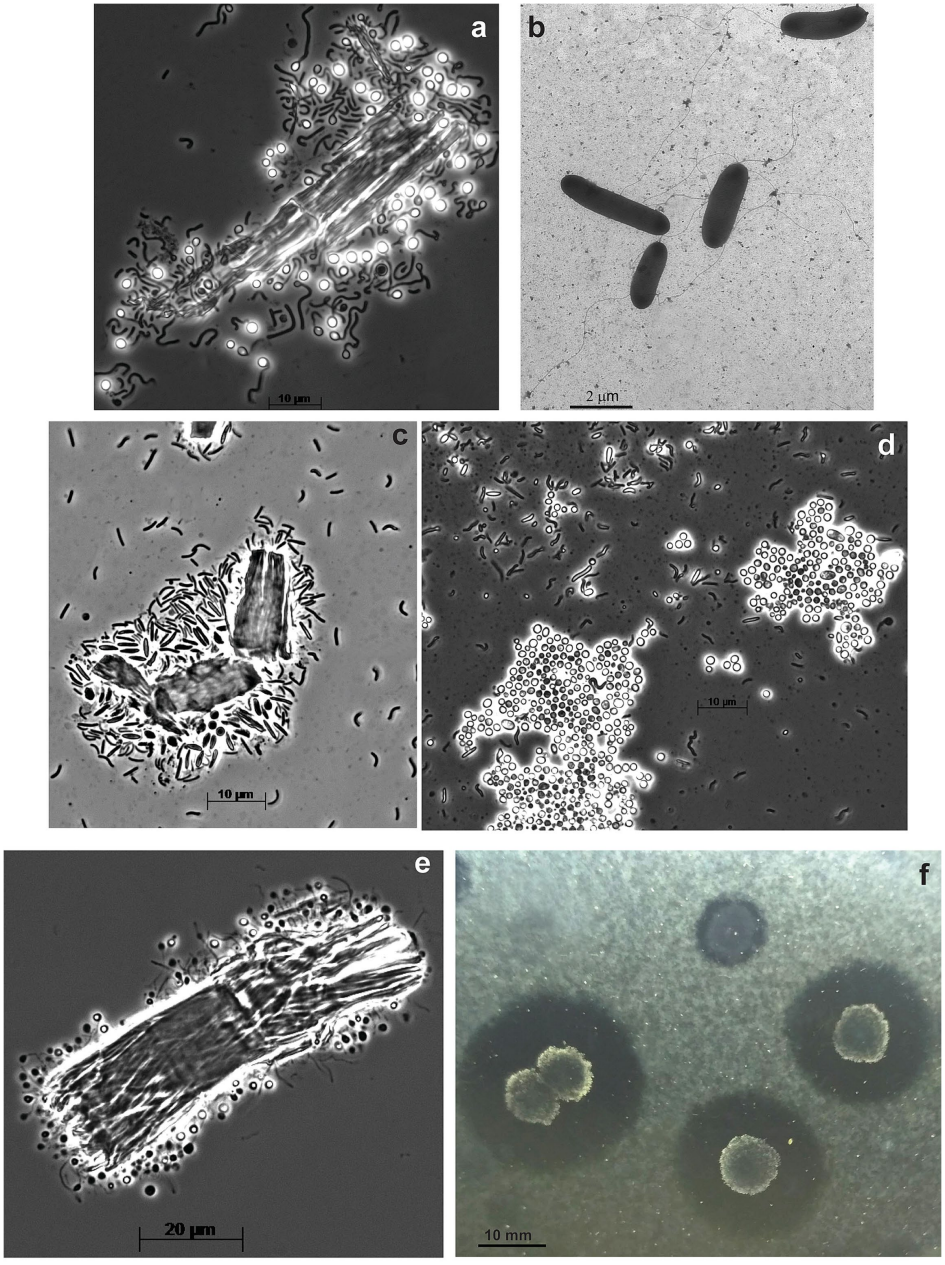
Cell and colony morphology of haloalkaliphilic anaerobic cellulotrophic bacteria isolated from soda lakes. **(A,B)** Endospore-forming *Clostridiales* strain ANBcel31: **(A)** colonizing Sigma cellulose 101 fragments and **(B)** free cells growing on cellobiose. **(C,D)** *Fibrobacterota* strain ABcell5: **(C)** young cells colonizing cellulose particles and **(D)** massive lipid cyst formation during growth on amorphous cellulose. **(E,F)** endospore-forming *Halanaerobiales* strain ANBcel28: **(E)** colonization of a cellulose fibril fragment; **(F)** soft agar colonies with amorphous cellulose hydrolysis zones.

The anaerobic enrichment on ac was finally resolved into three components by using the combination of serial dilution on liquid medium with ac followed by a soft ac-agar approach. The primary cellulotrophic bacterium in this culture formed white colonies with ac-clearance zones around and those colonies grew back in liquid culture with all 3 forms of cellulose used in this study and was designated as strain ANBcel5 (Supplementary Figure S2). The bacterium was identified as a member of *Fibrobacterota* by the 16S rRNA gene sequencing. It has vibrio to loose spirilla cells motile with a polar flagellum and formed abundant lipid cysts in aged cultures (Figures 3C,D), similar, but much more abundant, than in its closest chitin-utilizing relative *Chitinispirillum alkaliphilum* (Sorokin et al., 2016). The consortium also included two other fermentative anaerobes forming pink and yellowish colonies. The former is a long flexible non-motile rod (strain ANBcel2), while the latter has short bean-like motile cells (strain ANBcel3) (Supplementary Figures S3C–F). Both did not grow with cellulose in a liquid medium and were isolated in pure culture with cellobiose. The 16S rRNA gene sequencing identified strains ANBcel2 and ANBcel3 as members of the orders *Marinilabiliales* and *Balneolales*, respectively, in the *Bacteroidota* phylum.

The anaerobic enrichment on Sigma 101 cellulose was dominated by endospore-forming “worm”-like rods massively attacking cellulose fragments (Supplementary Figure S1C). Further serial dilution, after pasteurization for 10 min at 90°C, resulted in the isolation of the spore-forming cellulotrophic component in pure culture, designated strain ANBcel31, while plating on cellobiose (without pasterization) yielded yellow-colored colonies from which a saccharolytic satellite strain ANBcel1 unable to grow on cellulose was obtained in pure culture. The 16S rRNA gene sequencing identified cellulotrophic ANBcel31 as a distinct new lineage in the genus *Herbivorax* of the *Clostridiales* order, while the satellite saccharolytic strain ANBcel1—as a member of the order *Balneolales*, similar, but not identical to strain ANBcel3. ANBcel31 grew on all three used cellulose forms in a similar manner: initially, the cells massively colonized cellulose fragments, which turned bright yellow (Supplementary Figure S2). Then, free motile cells started to accumulate in the cellulose-free top medium layer (Figures 3A,B). The satellite strain ANBcel1 is a motile straight rod with pointed ends (Supplementary Figures S3A,B).

The anaerobic enrichment on fp looked very similar to the one on Sigma 101 cellulose with twisted endospore-forming rods abundantly colonizing cellulose fibers and with yellow-orange coloration (Supplementary Figure S3; Figure 3E). However, it did not grow back after the pasteurization of a maximal positive dilution. Plating on ac showed two types of colonies: large furry balls with cellulose clearance around (Figure 3F) and smaller colorless discs usually close to the cellulose-degrading colonies. The 16S rRNA sequencing from cells in the cellulose-degrading colonies produced a clean signal, and the organism (designated as ANBcel28) was identified as a distinct member of the order *Halanaerobiales* with *Halocella cellulolytica* as the closest validly published relative (Supplementary Figure S4), a halophilic anaerobic cellulotroph from the salt lake Sivash, a hypersaline lagoon of the Aral Sea (Simankova et al., 1993). Since multiple attempts to grow the cellulotroph from pure colonies in liquid cultures with various celluloses and cellobiose failed, the satellite bacterium from colorless colonies was obtained in pure culture on cellobiose (identified as *Natronincola*, also in *Clostridiales*). Then, the filter-sterilized culture supernatant and cell-free extract (obtained by sonication) were added to the liquid culture on ac inoculated with material from pure colonies of ANBcel28. However, this still did not allow the conspicuous bacterium to grow in liquid culture alone. Thus, the nature of its obligate syntrophy with the cellobiose-fermenting satellite remained unclear.

The comparative key phenotypic properties of the cellulotrophic isolates and their satellites are given in Table 1.

**Table 1:** Key phenotypic properties of cellulotrophic bacteria and their saccharolytic satellites obtained from cellulose enrichments from Siberian soda lakes.

Property	Aerobic		Anaerobic					
	ABcell2	**ABcell3**	**ANBcel5**	**ANBcel31**	**ANBcel28**	ANBcel2	ANBcel1	ANBcel3
Taxonomy*	*Cellvibrionales*	*Cytophagales*	*Chitivibrionales*	*Clostridiales*	*Halanaerobiales*	*Marinilabiales*	*Balneolales*	
Cell morphology								
Shape	Curved motile rods	Flexible rods	Motile vibrio	Plump rods	Flexible rods	Flexible rods	Motile rods	Motile curved rods
Size (μm)	1-4 x 0.4-0.5	3-12 x 0.2-0.4	1.5-5 x 0.3-0.4	2-6 x 0.7-1.0	2-12 x 0.1-0.15	2-6 x 0.15	1.5-2.0 x 0.5	1.5-3.0 x 0.5
Cyst like cells	−	+	+	−	−	−	−	−
Endospores	−	−	−	+	+	−	−	−
Pigmentation	Yellowish	-	-	Yellow-orange	Yellow	Pink	Yellowish	Yellowish
Growth with:								
Cellulose	−	**+**	**+**	**+**	**+**	−	−	−
Barley β-glucan	+	-	w+	−	+	−	−	−
Xyloglucan	+	w+	−	+	−	−	+	w+
Lichenan	+	−	+	−	+	−	+	−
Laminarin	+	−	−	+	−	+	+	−
Glucomannan	+	−	−	−	−	+	+	+
Beta-mannan	−	−	−	−	w+	−	−	−
Xylan	+	−	−	+	+	+	+	w+
Cellobiose	+	w+	+	+	nd	+	+	+
Starch	+	−	−	**−**	−	+	+	w+
Microaerobic growth**	+	+	−	−	−	−	+	−
H_2_ formation on cellulose/cb	nd	nd	−	+	+	Trace	−	−

Cellulotrophs are in bold, *Based on complete 16S rRNA gene sequences; ** statically at 2% O_2_ in the gas phase; nd – not detected.

### Functional genome analysis of extracellular glycosyl hydrolases

The main genomic property of the four cellulotrophic isolates from soda lakes is the large representation of the cellulase family GH9 and several subfamilies of the GH5, in contrast to their saccharolytic satellites (Table 2). Furthermore, in the two endospore-forming anaerobic cellulotrophs, the majority of the cellulases and other endo-beta-glucanases are apparently assembled into cellulosomal scaffolds as evidenced by the presence of dockerin domain as well as large cellulosome scaffold proteins containing multiple cohesion domains and S-layer homology (SLH) proteins (Artzi et al., 2017; Aikawa et al., 2018). The second in abundance in four cellulotrophs are several GH families of endo-beta-1,4 and 1,3/1,4 glucanases with putative activity against lichenan, glucomannan, and laminarin. Xylanase families GH10 and 11 are also abundantly represented in genomes of the endospore-forming ANBcel28 and ANBcel31. In contrast, the genomes of saccharolytic satellites mostly encode a few extracellular beta-glucanases (aerobic ABcell2 and anaerobic ANBcel1), and beta-glucosidases potentially enabling the utilization of xylan, laminarin, lichenan, glucomannan, and their oligomers and alpha-amylases/alpha-glucosidases for starch/pullulan depolymerization.

**Table 2:** Representation of excreted (sec/SPI-SPII or type IX secretion) glycosyl hydrolases in the genomes of soda lake cellulotrophic bacteria and their saccharolytic satellites.

GH family	Potential substrates	Primary cellulotrophs				Saccharolytic satellites			
		ABcell3	ANBcel5	ANBcel31	ANBcel28	ABcell2	ANBcel1	ANBcel2	ANBcel3
9	Cellulose	5(0*)	6(0*)	12(8*)	10(9*)	4(0*)	2(0*)	2(0*)	1(0*)
5_1/5-2		5	6	5*	3*	0	0	0	0
8	Cellulose, lichenan	4	1	1*	2*	0	0	0	0
5_4//5_7/5_8/5_10, 5_25/5/55; 26	Glucomannan	0	3	2*	3*	6	2	0	0
5_46; 16_3; 16_5; 16_21; 17; 81; 154 158	Laminarin	2	2	2*	1	2	3	4	0
16_25; 32; 148	Lichenan	3	2	2*	0	1	1	0	0
12; 31_3; 74	Xyloglucan	0	0	1*	2(1*)	0	2	0	2
10; 11; 30	Xylan	1	2	9(7*)	5(0*)	2	1	4	3
2; 3	Beta-glucosides	3	0	2(1)*	4(2*)	1	11	3	4
13(multiple subfamilies); 31; 92	Starch/pullulane	0	0	0	0	3	12	6	1

* Connected to the cellulosome scaffolds via dockerins.

### Phylogenomic placement of haloalkaliphilic cellulotrophic bacteria and their saccharolytic satellites

**Figure 4:**
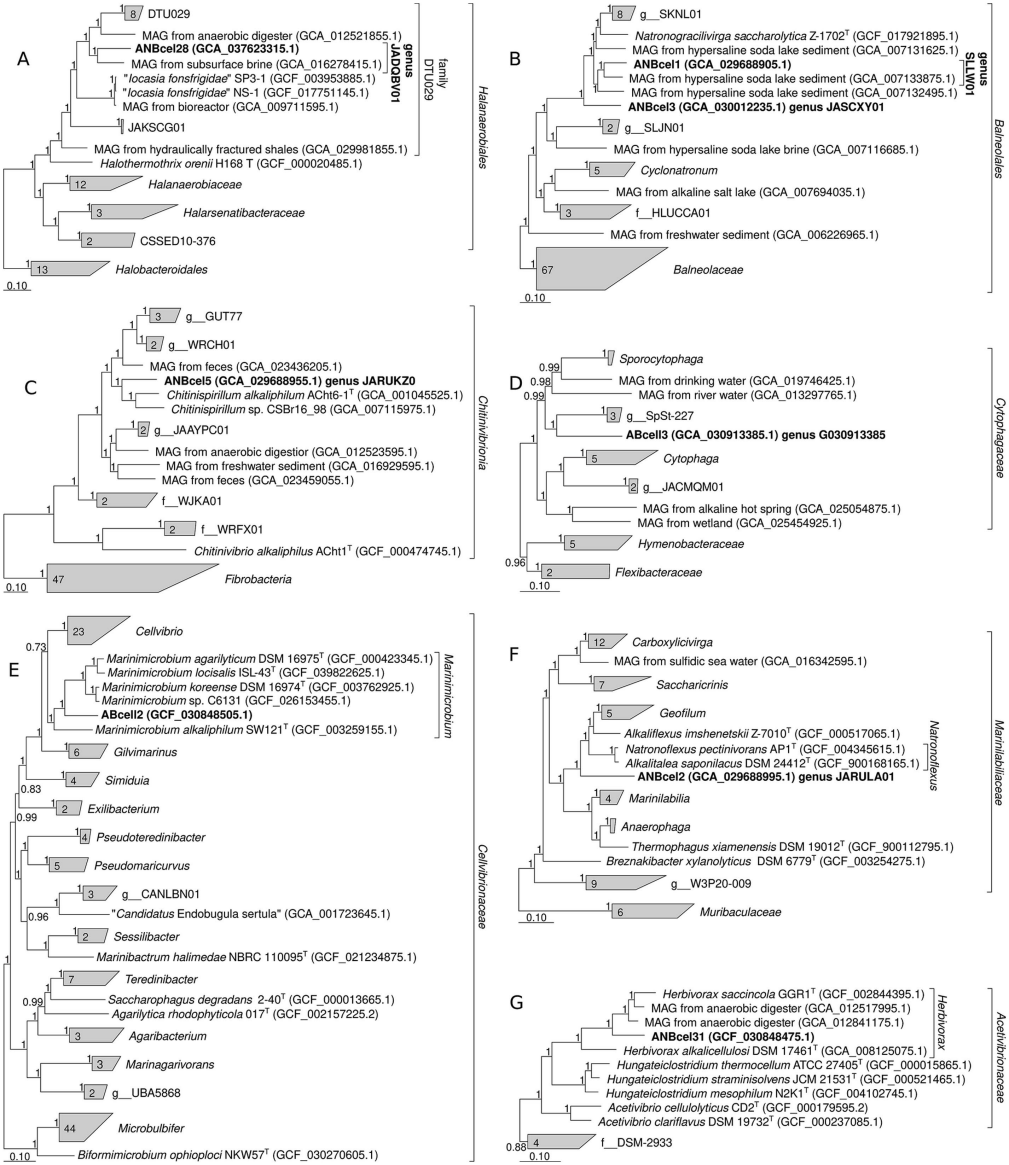
Phylogenetic position of haloalkaliphilic bacteria from soda lakes involved in cellulose mineralization based on concatenated amino acid sequences of 120 bacterial single-copy conserved marker proteins. **(A)** anaerobic cellulotrophic strain ANBcel28 within the order *Halanaerobiales* (class *Halanaerobiia*); **(B)** fermentative saccharolytic satellites of anaerobic cellulotrophs strains ANBcel1 and ANBcel3 within the order *Balneolales* (class *Rhodothermia*); **(C)** anaerobic cellulotrophic strain ANBcel5 within the class *Chitinivibrionia* (phylum *Fibribacterota*); **(D)** aerobic cellulotrophic strain ABcell3 within the family *Cytophagaceae* (class *Bacteroidia*); **(E)** aerobic hemicellulolytic satellite of cellulotrophic strain ABcell2 within the family *Cellvibrionaceae* (class *Gammaproteobacteria*); **(F)** anaerobic cellulotrophic strain ANBcel2 within the family *Marinilabiliaceae* (class *Bacteroidia*); and **(G)** anaerobic cellulotrophic strain ANBcel31 within the order *Acetivibrionaceae* (class *Clostrodia*). Taxonomic designations correspond to the Genome Taxonomy DataBase Release 09-RS220. The length of the alignment is 22,097 aa. Consensus branch support values from UFBoot and SH-like aLRT are shown at the nodes. Bars, 0.1 change per position.

Phylogenomic analysis based on the alignment of 120 single-copy conserved bacterial marker proteins confirmed that the aerobic cellulotrophic strain ABcell3 belongs to the order *Cytophagales* as a novel genus lineage (Figure 4D). The closest culturable relative to this strain is the soil cellulotroph *Sporocytophaga myxococcoides* (90.0% of 16S rRNA gene identity and 61.6% of AAI).

The aerobic hemicellulolytic satellite strain ABcell2 formed a distant new species in the genus *Marinimicrobium* within *Cellvibrionaceae* family (*Gammaproteobacteria*) (Figure 4E). By AAI analysis, the closest (70.8%) to this strain is the type species of the genus—*Marinimicrobium koreense* (Lim et al., 2006), whereas by 16S rRNA gene identity, the closest is *Marinimicrobium locisalis* (96.5%). The high salt-tolerant *Marinimicrobium* species and its nearest related members of the genus *Cellvibrio* are well known for their potential to utilize cellulose and other glucans (Moller et al., 2010; Tóth et al., 2021).

The phylogenomic analysis placed the anaerobic cellulotrophic strain ANBcel5 into the family *Chitinispirillaceae* (order *Chitinivibrionales* in the *Fibrobacterota*) as a new-genus lineage (Figure 4C). The closest cultured relative of ANBcel5 is the soda lake obligate chitinotroph *Chitinispirillum alkaliphilum* ACht6-1^T^ (Sorokin et al., 2016) with 95.2% of 16S rRNA gene identity and 64.4% of AAI. The RED value for the cluster combining the genus *Chitinispirillum* and ANBcel5 is 0.847, which is significantly lower than the median value of 0.922 for bacterial genera (according to GTDB release 09-RS220). The saccharolytic satellites of ANBcel5—strains ANBcel2 and ANBcel3 fell into families *Marinilabiliaceae* and *Cyclonatronaceae* (‘Natronogracilivirgulaceae’ according to GTDB release 09-RS220), respectively, each representing a new genus-level lineage (Figures 4B,F, respectively). The closest culturable relative to the strain ANBcel2 is the soda lakes anaerobic pectinolytic *Natronoflexus pectinivorans* (Sorokin et al., 2011). They have 92.1 and 91.45% of 16S rRNA gene identity and 67.1 and 67.2% of AAI, respectively. The closest culturable relative to the strain ANBcel3 is *Natronogracilivirga saccharolytica* (Zhilina et al., 2023): 94.8% of 16S rRNA gene identity and 67.9% of AAI. *N. saccharolytica* are the closest culturable microorganism to another saccharolytic satellite strain ANBcel1: 96.83% of 16S rRNA identity and 71.1% of AAI. Strains ANBcel3 and ANBcel1 have 95.9% of 16S rRNA gene identity and 68.2% of AAI to each other. Despite the fact that these three soda lakes saccharolytic anaerobes (*N. saccharolytica*, ANBcel1, and ANBcell3) formed a monophyletic group, they cannot be merged into one genus, because the cluster that they share has a RED value significantly lower than the median value for bacterial genera (0.839–0.863).

The cellulotrophic partner of the strain ANBcel1, strain ANBcel31, has been identified as a new species within the clostridial genus *Herbivorax* (Figure 4G), most closely related to the type species *H. saccincola* (Koeck et al., 2016). They have 95.3% of 16S rRNA gene identity and 76.7% of AAI. This genus is a member of the clostridial family *Acetivibrionaceae,* which also includes the genus *Hungateiclostridium*. The majority of the species in this family have been described as anaerobic cellulose and hemicellulose utilizers forming cellulosomes (Aikawa et al., 2018; Minor et al., 2024; Zhang et al., 2018).

Finally, phylogenomic reconstruction placed the cellulotrophic anaerobe ANBcel28 as a new genus within *Halanaerobiales* order with *Halocella cellulolytica* (94.2% of 16S rRNA gene identity) and “*Iocasia fonsfrigidae*” (93.3% of 16S rRNA gene identity and 64.4% of AAI) (Supplementary Figure S4; Figure 4A) as closest cultivated species. Both of them have been described as cellulotrophic endospore-forming fermentative anaerobes (Simankova et al., 1993; Zhang et al., 2021).

## Concluding remarks

This study demonstrated that native insoluble celluloses can be readily mineralized at moderately saline and highly alkaline conditions of soda lakes, supporting the growth of primary cellulotrophic haloalkaliphic bacteria and their saccharolytic satellites. Interestingly, the phylogenetic diversity of culturable anaerobic cellulotrophs in soda lakes appears to be higher than at aerobic conditions, hinting at a conclusion that cellulosic material is mostly mineralized in anoxic sediments in such systems. We cannot speculate on what might be found in other soda lakes, as each one differs in its physico-chemical composition and conditions. However, based on our findings, we can safely conclude the following: (1) to successfully select for specialized cellulotrophic microbes, it is necessary to use native forms of insoluble cellulose as the primary enrichment substrate, and (2) truly specialized cellulotrophs possess multiple representations of cellulases from the GH families 5 and 9. Given this, and considering the phylogenetic positioning of the cellulotrophs identified in this study, attempts could be made to identify potential candidates in metagenomes from soda lakes in other geographic areas.

The results also have implications for practical applications. For example, there is a large amount of toilet paper screened out in domestic wastewater treatment plants, where it is typically burned after recovery. Instead, it could be used to produce biogas with a high percentage of methane content, while the bulk CO_2_ is retained in the alkaline solution.

## Data Availability

The datasets presented in this study can be found in online repositories. The names of the repository/repositories and accession number(s) can be found at: https://www.ncbi.nlm.nih.gov/genbank/, BioProject PRJNA949680.

## Footnotes

^1^ https://github.com/donovan-h-parks/PhyloRank
