# Supplementary material for: Isolation and characterization of cellulose-mineralizing haloalkaliphilic bacteria from Siberian soda lakes
Source: Front Microbiol. 2024 Dec 23;15:1523074. doi: 10.3389/fmicb.2024.1523074 (PMC11700989; doi:10.3389/fmicb.2024.1523074)
Supplement: Supplementary file 1 [file Data_Sheet_1.PDF]

Isolation and characterization of cellulose-mineralizing haloalkaliphilic bacteria from Siberian soda lakes

Dimitry Y. Sorokin[1,2*], Alexander Y. Merkel[1] and Tatjana V. Khizhniak[1]

Supplementary data

Fig.S1 Bacterial colonization of cellulose fragments in primary enrichments from soda lakes. (**a**) aerobic enrichment on filter paper; (**b-c-d**) anaerobic enrichments of amorphous cellulose, Sigma 101 cellulose and filter paper, respectively.

Fig.S2 Growth of anaerobic haloalkaliphilic cellulotrophic bacteria with various forms of cellulose (*Clostridiales* ANBcel31 and *Halanaerobiales* ANBcel28) and growth dynamics with amorphous cellulose in *Fibrobacterota* ANBcel5. The mineral medium contained 1 M total Na^+ as sodium carbonates at pH 9.5. **Fp** – meshed filter paper.

Fig.S3 Cell morphology of haloalkaliphilic fermentative saccharolytic bacteroidetes isolated from soda lake anaerobic cellulotrophic consortia. (**a-b**) strain ANBcel1; (**c-d**) strain ANBcel3; (**e-f**) strain ANBcel2. The cultures were grown with cellobiose at 1 M total Na^+, pH9.5.

Fig.S4 Phylogenetic position of anaerobic cellulotrophic stain ANBcel28 within the order *Halanaerobiales* based on alignment of the 16S rRNA gene sequences. Consensus branch support values are shown at the nodes. Bar, 0.1 change per position.

Table S1. Genome statistics of cellulotrophic bacteria and their saccharolytic satellites obtained from cellulose enrichments from Siberian soda lakes.

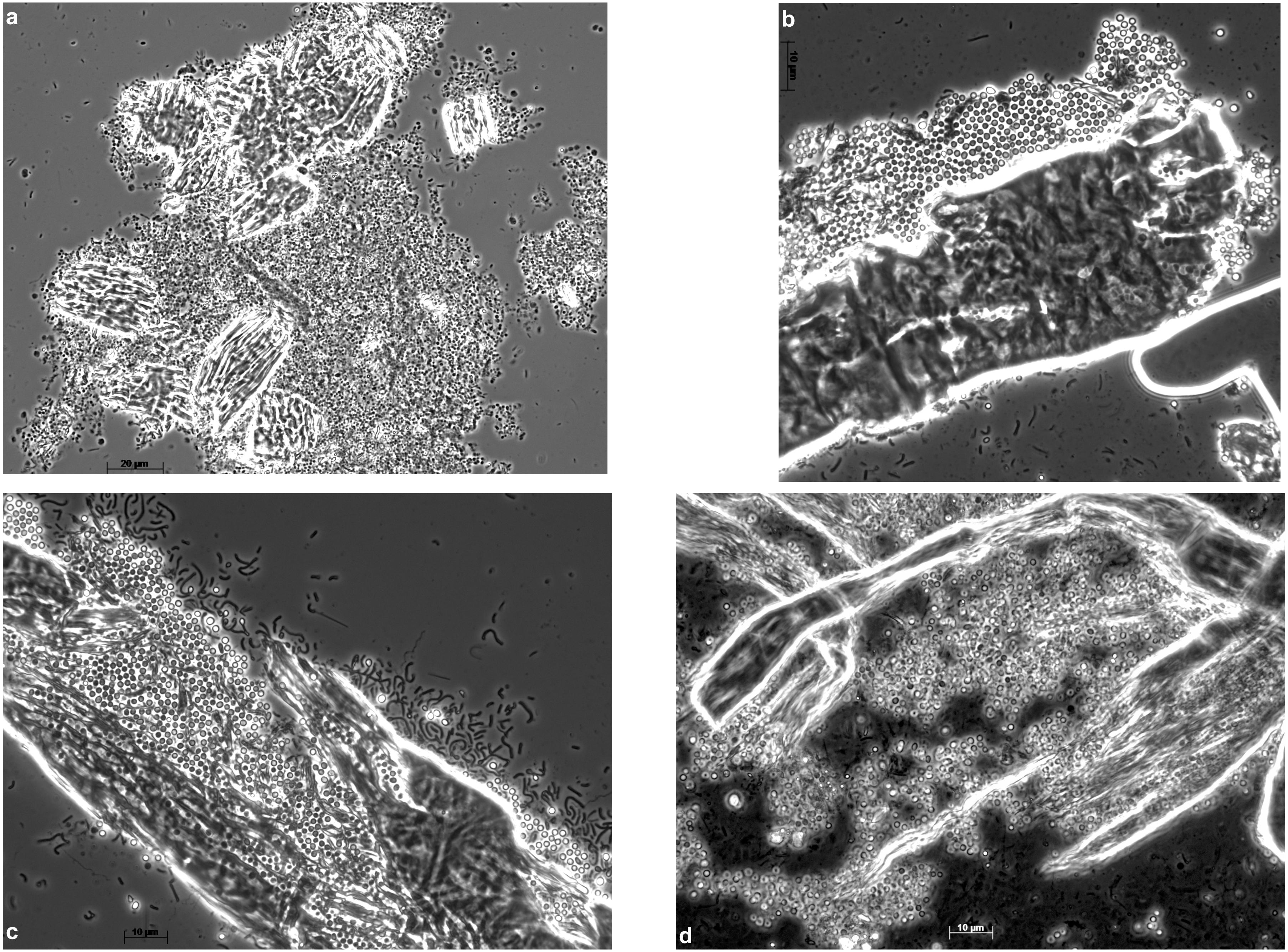

Fig.S1

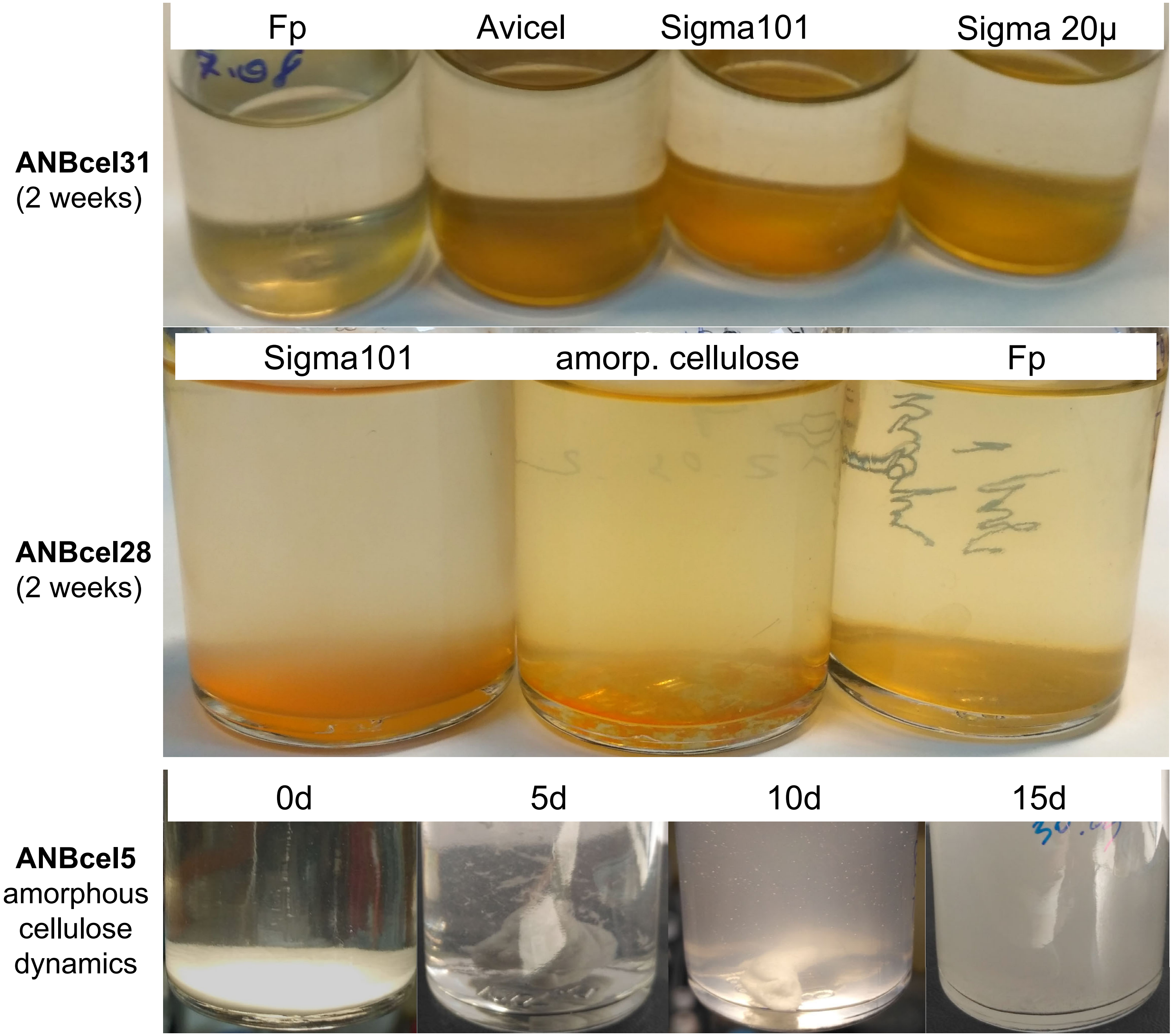
ANBcel31
(2 weeks)
Fp
Avicel
Sigma101
Sigma 20μ
ANBcel28
(2 weeks)
Sigma101
amorp. cellulose
Fp
ANBcel5
amorphous
cellulose
dynamics
0d
5d
10d
15d

Fig.S2.

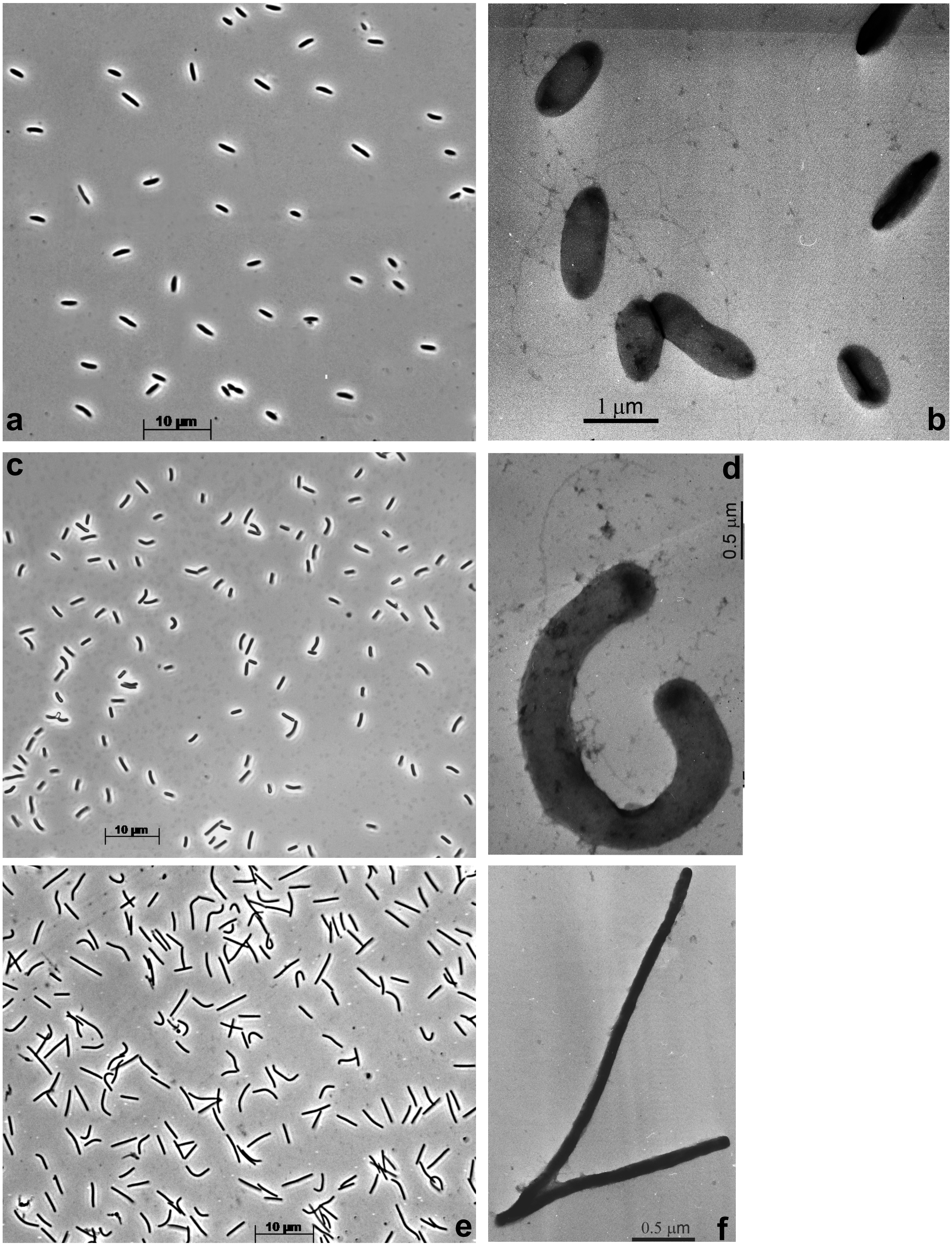

Fig.S3

Fig.S4

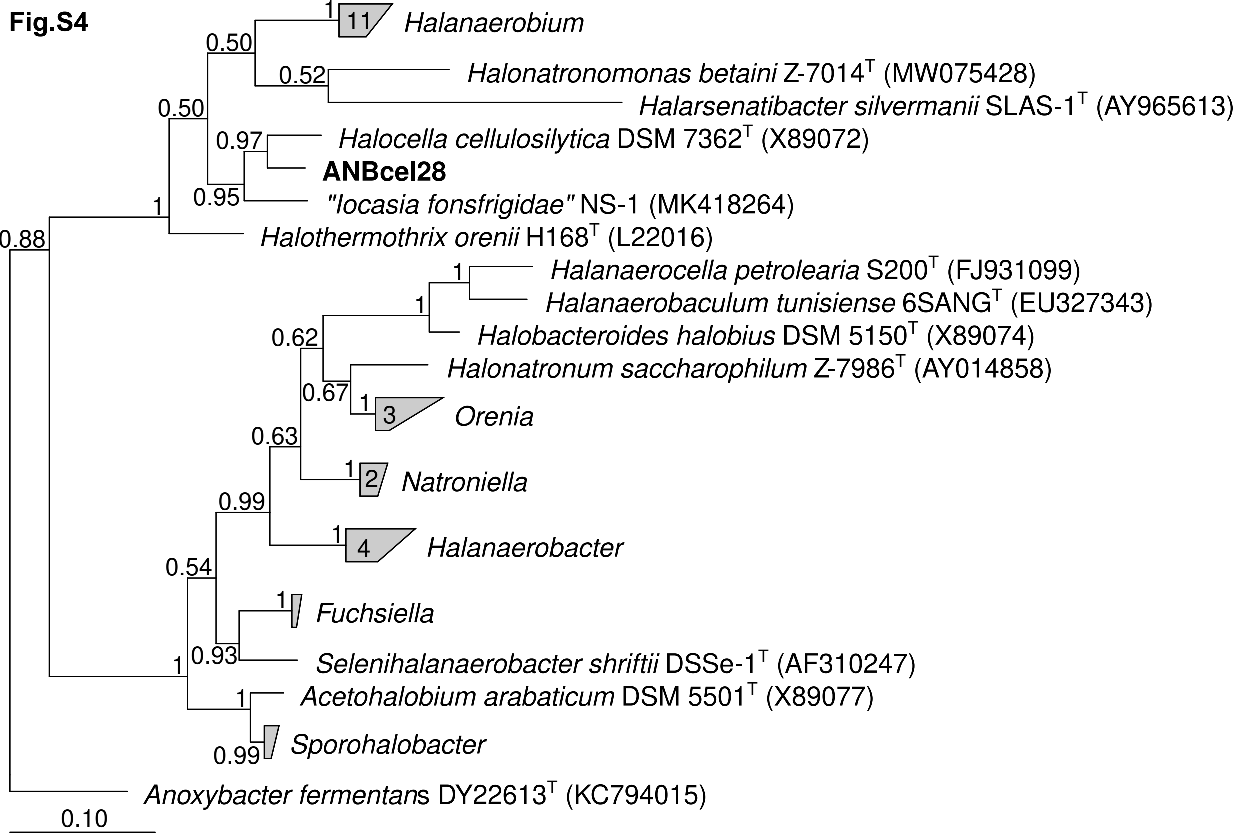

Halanaerobium
11
1
0.50
Halonatronomonas betaini Z-7014T (MW075428)
0.52
Halarsenatibacter silvermanii SLAS-1T (AY965613)
0.50
Halocella cellulosilytica DSM 7362T (X89072)
0.97
ANBcel28
"Iocasia fonsfrigidae" NS-1 (MK418264)
0.95
1
Halothermothrix orenii H168T (L22016)
0.88
Halanaerocella petrolearia S200T (FJ931099)
1
Halanaerobaculum tunisiense 6SANGT (EU327343)
1
Halobacteroides halobius DSM 5150T (X89074)
0.62
Halonatronum saccharophilum Z-7986T (AY014858)
0.67
1
3
Orenia
0.63
1
2
Natroniella
0.99
1
4
Halanaerobacter
0.54
1
Fuchsiella
0.93
Selenihalanaerobacter shriftii DSSe-1T (AF310247)
1
1
Acetohalobium arabaticum DSM 5501T (X89077)
Sporohalobacter
0.99
Anoxybacter fermentans DY22613T (KC794015)
0.10

Table S1. Key properties of genomes of cellulotrophic bacteria and their saccharolytic satellites obtained from cellulose enrichments from Siberian soda lakes.

Property	Aerobic		Anaerobic					
	ABcell2	ABcell3	ANBcel5	ANBcel31	ANBcel28	ANBcel2	ANBcel1	ANBcel3
Taxonomy	*Cellvibrionales*	*Cytophagales*	*Chitivibrionales*	*Clostridiales*	*Halanaerobiales*	*Marinilabiliales*	*Balneolales*	
Assembly level	Contig	Complete Genome	Contig	Contig	Scaffold	Contig	Contig	Contig
GenBank	GCA_030848505.1	GCA_030913385.1	GCA_029688955.1	GCA_030848475.1	GCA_037623315.1	GCA_029688995.1	GCA_029688905.1	GCA_030012235.1
Total length	4 Mb	5.1 Mb	4.7 Mb	4 Mb	3.7 Mb	3 Mb	3.3 Mb	3.2 Mb
Number of contigs	30	1	113	155	56	35	23	490
N50	423.9 kb	5.1 Mb	101.4 kb	68.2 kb	100.8 kb	278.2 kb	394.4 kb	13 kb
L50	4	1	15	19	12	3	4	69
GC percent	55	39.5	41.5	31.5	31.5	36	53.5	45
Completeness*	100%	100%	98.83%	99.38%	98.74%	99.46%	96.44%	97.54%
Contamination*	1.12%	2.18%	1.1%	0.78%	0.84%	0.54%	3%	4.1%
Genes (total)	3539	4212	3791	3561	3319	2398	2570	2845
Genes (protein coding)	3489	4124	3733	3465	3246	2341	2516	2795
rRNAs	1, 1, 1 (5S, 16S, 23S)	5, 5, 5 (5S, 16S, 23S)	1, 1 (16S, 23S)	1, 1 (16S, 23S)	4, 1, 1 (5S, 16S, 23S)	1, 1, 1 (5S, 16S, 23S)	1, 1, 1 (5S, 16S, 23S)	1, 1, 1 (5S, 16S, 23S)
tRNAs	41	47	41	46	50	43	41	38
Pseudo Genes (total)	2	24	12	44	13	8	7	6

* calculated using CheckM v1.2.3 (Parks, D. H., Imelfort, M., Skennerton, C. T., Hugenholtz, P., & Tyson, G. W. (2015). CheckM: assessing the quality of microbial genomes recovered from isolates, single cells, and metagenomes. Genome research, 25(7), 1043–1055. https://doi.org/10.1101/gr.186072.114).
